# Abnormal bowel movement frequency increases the risk of rectal cancer: evidence from cohort studies with one million people

**DOI:** 10.1042/BSR20200355

**Published:** 2020-04-15

**Authors:** Lin Shen, Chao Li, Na Li, Liangfang Shen, Zhanzhan Li

**Affiliations:** Department of Oncology, Xiangya Hospital, Central South University, Changsha, Hunan Province 410008, China

**Keywords:** bowel movement, cohort study, colorectal cancer, meta-analysis, risk factors

## Abstract

Previous studies from case–control studies failed to draw reliable conclusions regarding the relationship between bowel movement frequency and the risk of colorectal cancer. To further examine this relationship, we collect the data from cohort studies that make a more accurate estimation. Several online data were searched from inception to February 29, 2020. Ten cohort studies involving 1,038,598 individuals were included in our study. The pooled results indicated that a bowel movement of less than once per day was not associated with the risk of colorectal cancer (relative risk (RR)= 1.00, 95% confidence interval (CI): 0.87–1.16, *P* = 0.950) compared with that of once per day. Compared with a bowel movement frequency of once per day, a bowel movement of more than once per day was also not related to elevated risk of colorectal cancer (RR = 1.04, 95% CI: 0.91–1.19, *P* = 0.570). The subgroup analyses indicated a low or high bowel movement frequency did not increase the risk of colon cancer (RR = 0.91, 95% CI: 0.80–1.03, *P* = 0.130). However, an increased frequency of bowel movements increased the risk of rectal cancer (RR = 1.34, 95% CI: 1.19–1.52, *P* < 0.001). The sensitivity analysis still supports the results. No significant publication bias existed. The data from cohort indicated that less bowel movement frequency was not associated with the risk of colorectal cancer. The frequency of bowel movement affects the risk of rectal cancer.

## Introduction

Colorectal cancer is one of the most common cancers regardless of males and females [1]. There were more than 1.8 million new cases in 2018 [2]. Colorectal cancer has become a major health problem. Previous studies have identified some potential relevant factors of colorectal cancer, including consuming processed meat or red meat, consuming alcohol, smoking, obesity, etc. [3–6]. The occurrence of colorectal cancer involves many complex molecular mechanisms [7–9]. Defecation is the final stage of food passing through the digestive tract. Bowel movement frequency and fecal characteristics are indicators of intestinal health and can also reflect the status of intestinal flora to some extent [10]. Relatively low or high bowel movement frequencies may be associated with colon cancer risk [11,12]. A previous meta-analysis based on nine case–control studies suggested that infrequent bowel movements or constipation significantly were associated with the risk of colorectal cancer. In contrast, a cohort study did not support such a relationship [13]. Besides, the most recent meta-analysis of case–control studies (in 2013) indicated that constipation was significantly associated with colorectal cancer. However, similar results were not found in three cohort studies [14]. The above studies had some methodological limitations, such as retrospective design bias, inconsistent definitions of constipation, difference in adjusting confounding factors and the use of prospective data. Recently, several new cohort studies have reported the association between bowel movement frequency and colorectal cancer, but these data have also yielded inconsistent results [14,15]. To further examine the association between bowel movement frequency and risk of colorectal cancer, we collected data form the cohort studies including more than one million study participants and made more accurate estimates.

## Materials and methods

We performed this meta-analysis according to the Preferred Reporting Items for Systematic Reviews and Meta-analyses statement guidelines (Supplementary material 1). This is a meta-analysis based on previous studies and the ethic approval is not applicable. The included studies have declared that they were approved by the ethics committee of their own. The studies have been carried out in accordance with the World Medical Association Declaration of Helsinki, and that all subjects provided written informed consent.

### Search strategy

We performed search in the PubMed, EMBASE, Web of Science, EMBASE, CNKI and Wanfang databases from their inception to February 29, 2020. The corresponding search terms mainly consisted of (colorectal cancer, colon cancer, rectal cancer, colorectal carcinoma, colon adenocarcinoma, rectal adenocarcinoma, bowel cancer, colon carcinoma, rectal carcinoma, colorectal adenocarcinoma, colonic neoplasms, rectal neoplasms, colorectal neoplasms, CRC) AND (bowel movement, bowel movement frequency, bowel habit, constipation) AND (prospective cohort study, retrospective cohort study, prospective, retrospective, cohort study). The search strategy is presented in Supplementary material 2. We also checked the references lists of some of the reviews for additional potentially eligible studies. The search languages were restricted to English and Chinese.

### Definitions of exposures and outcomes

The exposure of interest was frequency of bowel movement. Almost all studies considered the frequency of once per day as the control. Therefore, normal bowel movement was defined as once per day. Abnormal bowel movement was defined as less than once per day or more than once per day. The outcomes of interest were colorectal cancer, including colon cancer and rectal cancer.

### Study selection

Two investigators (L.Y.Y. and L.N.) performed the literature search, excluded duplicate studies, and scanned the titles and abstracts of studies, during which we intentionally expanded the search to include all potential studies. Finally, we reviewed the full texts of the selected studies for extracted data. The inclusion criteria included: (1) the study was a prospective or retrospective cohort study in a general population; (2) the study classified those with a bowel movement frequency of once per day as the unexposed group, and the rest were classified as the exposed group; and (3) the study provided sufficient data to calculate relative risks (RRs) and theirs 95% confidence intervals (CIs) for colorectal cancer, including the risk ratio and hazard ratio (HR). For studies with duplicated data, the most recent publication was used. Reviews, letters, comments, editorials, experiments and qualitative studies were excluded. Any disputes were resolved through discussion.

### Data extraction

The data extraction was performed by S.-L. and confirmed by other authors (Z.Q. and L.C.). For each study, we mainly extracted the following information: surname of the first author; publication year; country; type of study design (retrospective vs. perspective cohort); sample size; mean age or age range of study population; methods of bowel movement reporting (self-reported vs. others); follow-up period (year); colorectal cancer rates, including colon cancer and rectal cancer; adjusted factors in the multivariate analysis; risk ratios and 95% CIs in each study (adjusted and unadjusted); and events and nonevents in exposure group and control group.

### Quality assessment

We used the Newcastle–Ottawa Scale (NOS) (cohort study version) for quality assessments of included studies [16]. This scale has been extensively used in the assessment of cohort study and consists of three domains: selection, comparability and outcomes. Each item in the selection and outcome domains is assigned one score (one star). Comparability is assigned two scores (two stars). The lowest score was 0 and the highest was 9. Study with ≥6 scores will be considered high quality.

### Statistical analysis

We used the RR as the measurement parameter for the relationship between bowel movement and colorectal cancer risk. The hazard risk was directly regarded as the RR. The corresponding 95% CIs were also calculated [17]. We extracted the cases and controls in the exposed group and the unexposed group. For the adjusted data in the regression analysis, the risk estimations and 95% CIs were extracted from each study. The estimations and their standard errors were log- transformed for further analysis [6]. The heterogeneity within studies was assessed by the Cochran *Q* test and *I*^2^ statistic [18]. The Cochran *Q* test was a qualitative analysis, and *P* < 0.05 was considered significant heterogeneity. For the quantitative analysis, *I*^2^ ≤ 25% indicated low heterogeneity, 25% < *I*^2^ ≤ 50% for mild heterogeneity, 50% < *I*^2^ ≤ 75% for moderate heterogeneity, and *I*^2^ > 75% means high heterogeneity [19]. The heterogeneity is used for selecting random effects model or fixed-effect model. Subgroup analyses were performed based on cancer type. The sensitivity analysis was performed by two methods. We first compared the pooled results between the fixed-effect model and the random effects model, and then we performed the remaining pooled analyses by omitting a single study because the adjusted factors differed. Begg’s and Egger’s tests were used for publication bias assessment [20,21]. *P* < 0.05 indicated statistically significant differences. All analyses were performed using Stata 14.0 and Review Manager Version 5.3 software.

## Results

### Identification and characteristics of the included studies

**Figure 1:**
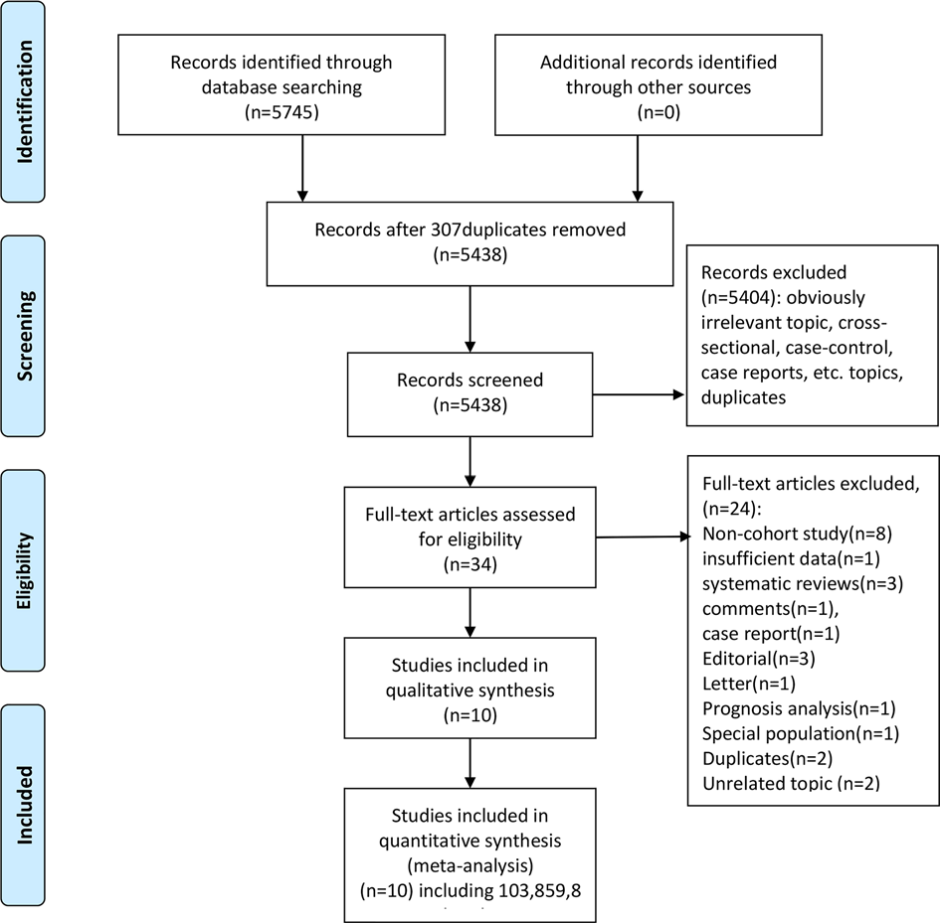
The flow chart of study selection

Initially, we obtained 5745 records identified through the database search. Of these, 307 duplicates were excluded, and 5438 records were further screened. We further excluded 5404 records with obviously irrelevant topics or that were cross-sectional or case–control designs, case reports or other types of publications. Thirty-four records were assessed for eligibility by reviewing the full-text articles. We excluded 24 studies, including eight non-cohort studies, one study with insufficient data, three systematic reviews, one comment, one case report, three editorials, one letter, one prognosis analysis, one study among a special population, two duplicates and two unrelated topics. The list of the excluded studies with the exclusion reasons are presented in Supplementary Material S3. Finally, ten cohort studies were included [15,22–30]. The study selection process is presented in Figure 1.

The Table 1 presents the general characteristics of these studies. These studies were published from 2000 to 2019. Three studies were from Japan, three were from the U.S.A., one was from the U.K., one was from China, one was from the Netherlands and one was from New Zealand. The ten studies included 1,038,598 study participants, including 9701 patients with colorectal cancer. The sample size ranged from 930 to 510,134 participants. One study included only people older than 18 years. The remaining studies were performed among populations whose ages ranged from 30 to 79 years. The bowel movement frequencies in all studies were based on self-reported data. The follow-up time was from 6 to13.3 years. The number of adjusted factors was different in each study; however, some common variables were included in the multivariate regression: age, gender, smoking, body mass index, meat intake, etc.

**Table 1:** General characteristics of included in the meta-analysis

Author	Year	Country	Study design	Sample size	Age (y)	Bowel movement	Follow-up year	Study period	Colorectal cancer	Colon cancer	Rectal cancer	Adjusted factors
Nakaya	2004	Japan	Prospective	41,670	40–64	Self-reported	7	1990–1997	251	156	95	Gender, age, smoking, alcohol, body mass index, education, family history of colorectal cancer, pork intake, green vegetables intake, orange, intake, walking time per day
Park	2009	U.K.	Prospective	930	45–79	Self-reported	12	1993–2005	159	–	–	Age, gender, BMI, WHR, smoking, energy intake, alcohol intake, dietary fiber, total meat intake
Otani	2006	Japan	Prospective	57,940	40–69	Self-reported	7.9	1990–2001	479	298	181	Age, Public Health Center areas, smoking, alcohol consumption, body mass index, physical exercise and refraining from animal fat
Kojima	2004	Japan	Prospective	62,929	40–79	Self-reported	9	–	649	429	220	Age, BMI, intake frequency of green leafy vegetables, daily alcohol drinking, current smoking status, time spent for walking per day, family history of colorectal cancer and education
Citronberg	2014	New Zealand	Prospective	75,214	50–76	Self-reported	6	2002–2008	396	294	102	Age, sex, race/ethnicity, BMI, physical activity, education, fiber, calcium, fruit and vegetable intake, red/processed meat intake, alcohol consumption, smoking, NSAID use, aspirin use, family history of CRC, colonoscopy / sigmoidoscopy screening, history of polyp removal, hormone replacement therapy use, and caloric intake, laxative use, fiber laxative use, bowel movement frequency and constipation
Simons	2010	Netherlands	Prospective	1753	55–69	Self-reported	13.3	1986–1999	687	449	238	Age, family history of colorectal cancer, occupational physical activity, nonoccupational physical activity, smoking status, alcohol intake, socioeconomic status, body mass index, fresh meat intake, processed meat intake, and dietary fiber intake
Yang	2019	China	Prospective	510,134	52 (mean)	Self-reported	9.9	2004–2016	3023	1548	1475	Age, gender, education, occupation, salary, marriage, history of cancer, smoking, physical, vegetables intake, meat intake, fruits intake, alcohol, MBI, WHR
Zhang	2013	U.S.A.	Prospective	88,173	40–75	Self-reported	11	2000–2010	2012	–	–	Age, smoking before age 30, history of colorectal cancer in a parent or sibling, history of endoscopy, regular aspirin use, body mass index, physical activity, postmenopausal hormone use, consumption of processed meat, alcohol consumption, energy-adjusted total calcium intake, total folate, total fiber, total vitamin D intake and total energy intake
Dukas	2000	U.S.A.	Prospective	84,439	30–55	Self-reported	12	1984-1996	611	485	126	Age, body mass Index, fiber intake, physical activity, postmenopausal, status and hormone use, and use of laxatives
Guerin	2014	U.S.A.	Prospective	115,416	Above 18	Self-reported	11	1999–2010	1434	–	–	Age, gender, family history of malignancies, and other physical and mental comorbidities with a prevalence of ≥5% in both the CC and CC-free cohorts and for which the difference at baseline was statistically significant (i.e. hypertension, diabetes mellitus without chronic complication, solid tumor without metastasis, any malignancy and chronic pulmonary disease

According to the NOS, these studies had generally good quality, with a mean of 8.3 points. The NOS scores ranged from 7 to 9. The details of each domain and item are presented in Supplementary Material S4.

### Bowel movement and risk of colorectal cancer

#### Frequency of less than one time per day versus once per day

**Figure 2:**
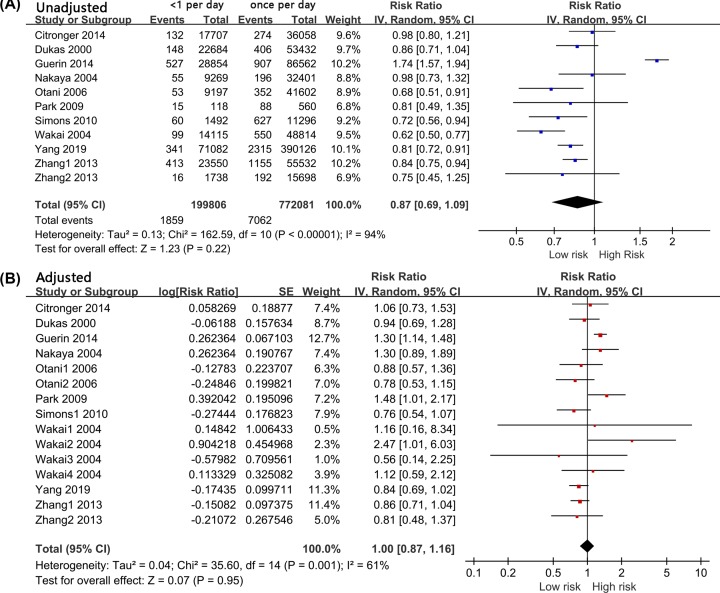
Forest Plot: Less than once a day versus once a day for the risk of colorectal cancer (**A**): unadjusted; (**B**): adjusted.

Eleven groups of data from ten studies reported comparison results between a frequency of <1 time per day and once per day. The random effect model was used because of the significant heterogeneity across the studies (*I*^2^ = 94%, *P* < 0.001). Compared with the group with a frequency of once per day, the group with a frequency of <1 time per day did not have an elevated risk of colorectal cancer (RR = 0.87, 95% CI: 0.69–1.09, *P* = 0.220, Figure 2A). We extracted 15 sets of data with adjusted potential cofounding factors from 10 studies. The results of the random effects model indicated that a frequency of less than one time per day did not increase the risk of colorectal (RR = 1.00, 95% CI: 0.87–1.16, *P* = 0.950, Figure 2B). A summary of the pooled results regarding the relationship between the frequency of bowel movements and colorectal cancer risk is presented in Table 2.

**Table 2:** Summary of pooling results about the relationship between bowel movement and colorectal cancer risk

Category	Group	Number of data group	RR	95% CI	Model	Overall effect test	Publication bias	
							Egger	Begg
**Unadjusted**								
<1 time per day	Colorectal cancer	11	0.87	0.69–1.09	Random	0.220	0.270	0.484
	Colon cancer	8	0.82	0.76–0.89	Fixed	0.001	0.095	0.216
	Rectal cancer	8	0.73	0.64–0.82	Fixed	0.001	0.153	0.458
>1 time per day	Colorectal cancer	7	1.03	0.88–1.20	Random	0.740	0.028	0.652
	Colon cancer	7	1.11	1.01–1.22	Fixed	0.030	0.190	0.293
	Rectal cancer	5	1.45	1.29–1.62	Fixed	0.000	0.053	0.027
**Adjusted**								
<1 time per day	Colorectal cancer	15	1.00	0.87–1.16	Random	0.950	0.605	0.299
	Colon cancer	11	0.91	0.80–1.03	Fixed	0.130	0.212	0.186
	Rectal cancer	8	0.93	0.78–1.11	Fixed	0.420	0.559	0.216
>1 time per day	Colorectal cancer	9	1.04	0.91–1.19	Random	0.570	0.314	0.677
	Colon cancer	6	1.04	0.92–1.18	Fixed	0.054	0.134	0.851
	Rectal cancer	6	1.34	1.19–1.52	Fixed	0.000	0.335	0.348

#### More than once per day vs. once per day

**Figure 3:**
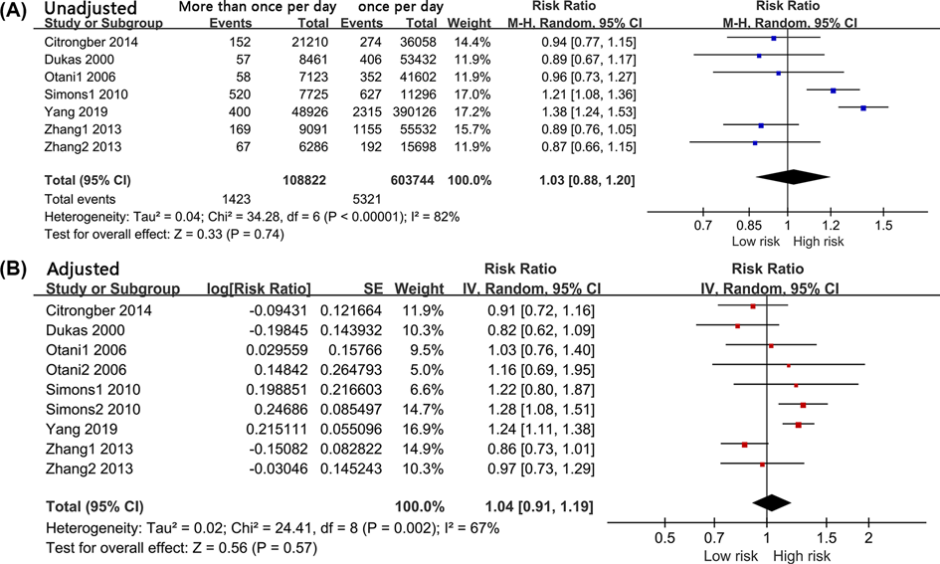
Forest Plot: More than once a day versus once a day for the risk of colorectal cancer (**A**): unadjusted; (**B**): adjusted.

Seven sets of data were used to assess the relationship between bowel movement of more than once per day and colorectal cancer risk. High heterogeneity was obtained (*I*^2^ = 82%, *P* < 0.001). Using a random effect model, we found that a bowel movement frequency of more than once per day was not associated with colorectal cancer occurrence compared with a frequency of once per day (RR = 1.03, 95% CI: 0.88–1.20, *P* = 0.740, Figure 3A). Nine sets of data were extracted for the adjusted analysis. There was moderate but significant heterogeneity (*I*^2^ = 67%, *P* = 0.002). Compared with a bowel movement frequency of once per day, the frequency of more than once per day was not related to colorectal cancer risk (RR = 1.04, 95% CI: 0.91–1.19, *P* = 0.570, Figure 3B).

### Subgroup analyses

#### Less than one time per day versus once per day

**Figure 4:**
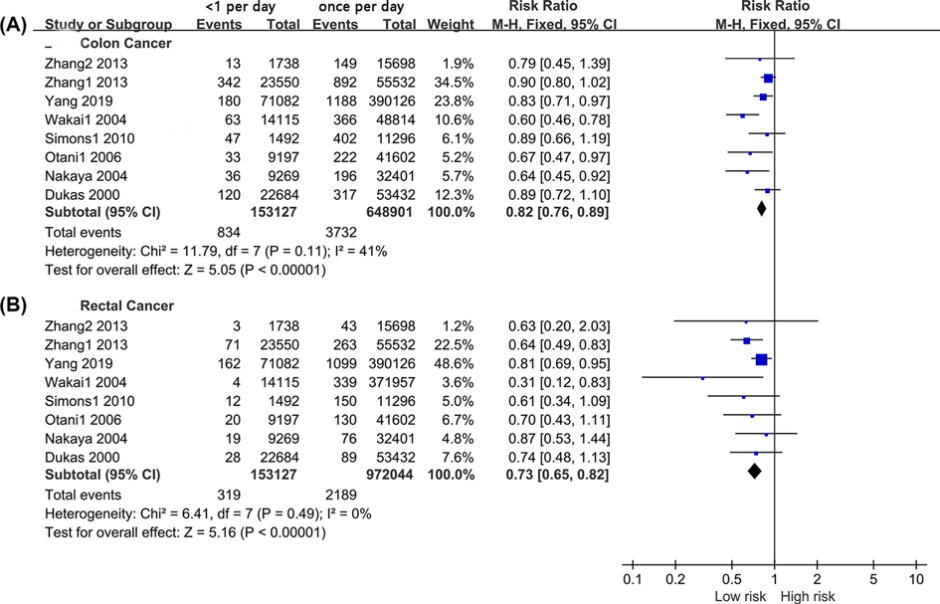
Forest Plot: The unadjusted relation of less than once a day with colon cancer and rectal cancer risk (**A**): colon cancer; (**B**): rectal cancer.

**Figure 5:**
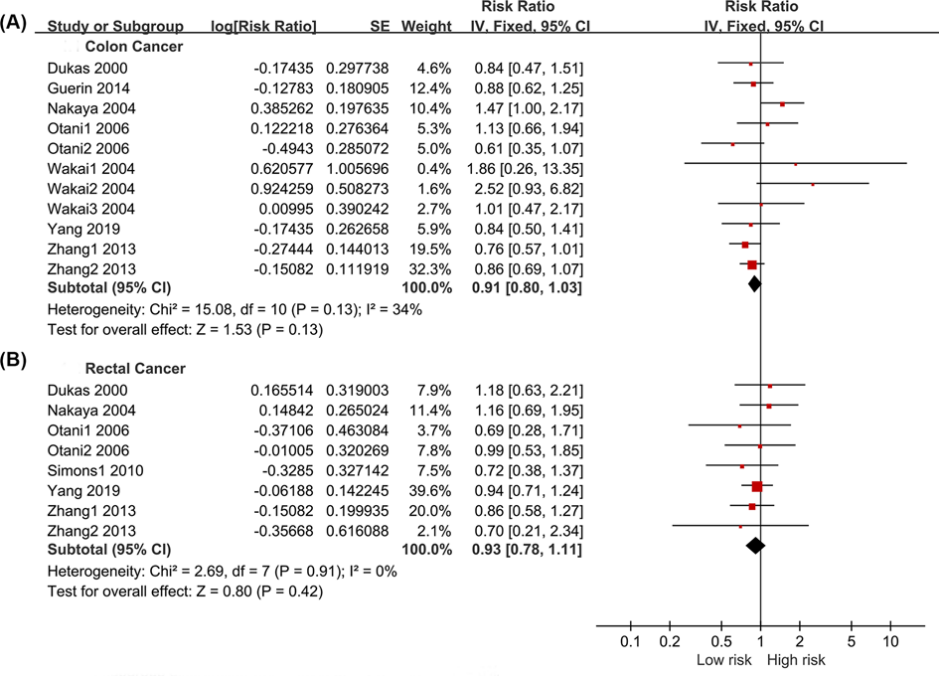
Forest Plot: The adjusted relation of less than once a day with colon cancer and rectal cancer risk (**A**): colon cancer; (**B**): rectal cancer.

Subgroup analyses were conducted based on cancer type (colon cancer and rectal cancer). We extracted eight sets of colon and rectal cancer data from ten studies. Mild heterogeneity was found in two subgroups, and fixed-effect models were used (*I*^2^ = 41% and *I*^2^ = 0%, respectively). The pooled results indicated that a bowel movement frequency of <1 day reduced the risks of colon cancer (RR = 0.82, 95% CI: 0.76–0.89, *P* < 0.001, Figure 4A) and rectal cancer (RR = 0.73, 95% CI: 0.65–0.82, *P* < 0.001, Figure 4B) compared with a frequency of once per day. For the adjusted data, there was also mild heterogeneity within the studies. The results from the fixed-effect model indicated that bowel movement frequency was not significantly related to colon cancer (RR = 0.91, 95% CI: 0.80–1.03, *P* = 0.130, Figure 5A) or rectal cancer (RR = 0.93, 95% CI: 0.78–1.11, *P* = 0.420, Figure 5B).

#### More than once per day versus once per day

**Figure 6:**
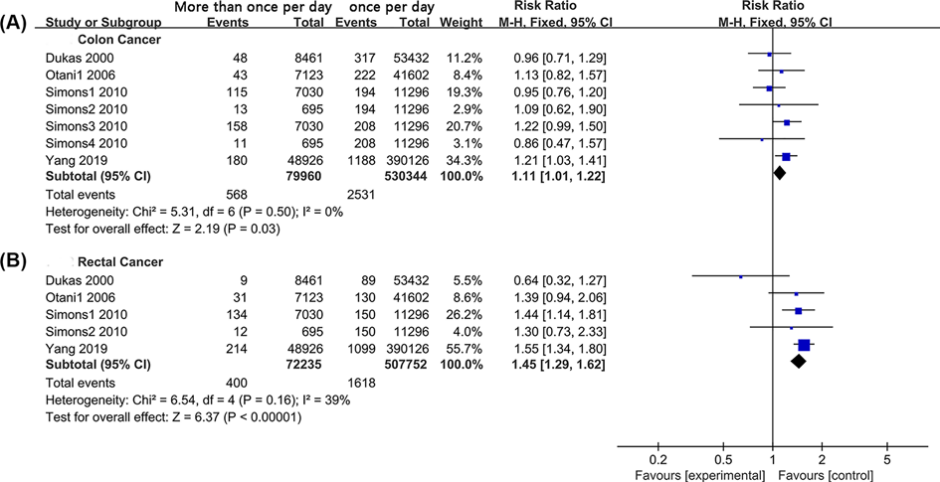
Forest Plot: The unadjusted relation of more than once a day with colon cancer and rectal cancer risk (**A**): colon cancer; (**B**): rectal cancer.

**Figure 7:**
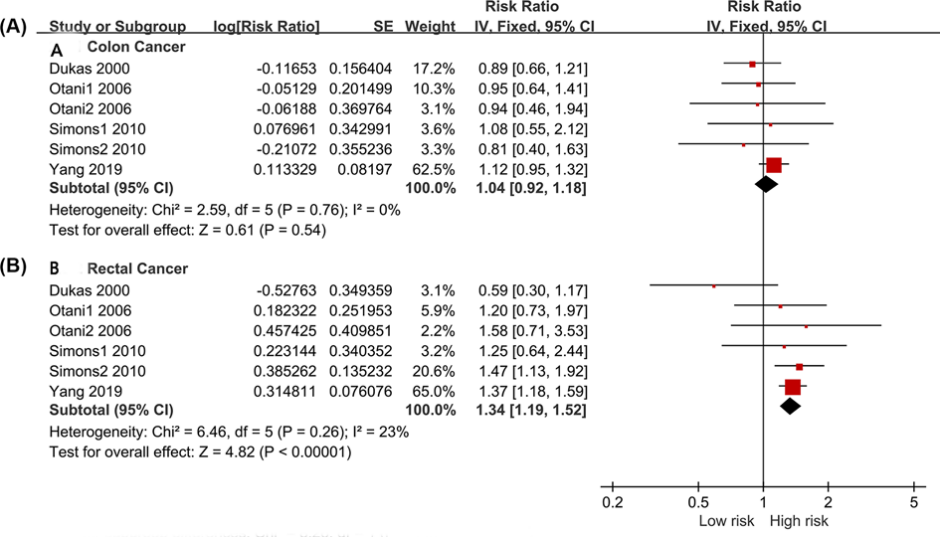
Forest Plot: The adjusted relation of more than once a day with colon cancer and rectal cancer risk (**A**): colon cancer; (**B**): rectal cancer.

We extracted seven sets of colon cancer data and five sets of rectal cancer data. The colon cancer and rectal cancer datasets included 610,304 and 1,229,987 study subjects. The pooled results of the fixed-effect model suggested that a bowel movement frequency of more than once per day increased the risks of colon cancer (RR = 1.11, 95% CI: 1.01–1.22, *P* = 0.030, Figure 6A) and rectal cancer (RR = 1.45, 95% CI: 1.29–1.62, *P* < 0.001, Figure 6B) compared with a frequency of once per day. Furthermore, the adjusted data were analyzed. Six sets of colon cancer and rectal cancer data were extracted. There was no significant heterogeneity within the studies (*I*^2^ = 0% and *I*^2^ = 23%, respectively). The results indicated a non-significantly relationship between bowel movement frequency of more than once per day and the risk of colon cancer (RR = 1.04, 95% CI: 0.92–1.18, *P* = 0.540, Figure 7A). However, an increased frequency of bowel movements increased the risk of rectal cancer (RR = 1.34, 95% CI: 1.19–1.52, *P* < 0.001, Figure 7B).

### Sensitivity analyses

We first compared the results of the fixed-effect model and random effects model to identify the influence of small-study effects on the pooled data. The estimations from the two methods were similar (Supplementary Material S5). Then, an analysis of the influence of a single study was performed by omitting one study at a time. The results remained stable (Supplementary Material S6).

### Publication bias

All the results of the publication bias analyses, including the overall pooled and subgroup analyses, are presented in Table 2. All Egger’s test results indicated no publication bias (*P* > 0.05); but significant publication bias was found according to the unadjusted data of rectal cancer (>1 time per day).

## Discussion

The present meta-analysis comprehensively and systematically reviewed the data from cohort studies and found the following: (1) Overall, the unadjusted and adjusted pooled results indicated that a bowel movement frequency of <1 time per day was not associated with colorectal cancer compared with a frequency of once per day. (2) Compared with once per day, a bowel movement frequency of >1 time per day was also not related to colorectal cancer risk. (3) However, the subgroup analyses indicated that the frequency of >1time once per day increased the risk of rectal cancer but not colon cancer after adjusting for potential factors. The present study contained more than one million study participants and high-quality cohort study data, providing strong evidence supporting the association between bowel movement frequency and colorectal cancer risk.

Two meta-analyses based from case–control studies have been published. The main findings were inconsistent with our pooled results. The differences between our study and the previous studies should be noted. First, the previous studies combined the data from case–control studies, while our study consisted of only cohort studies. Cohort studies better illustrate causal relationships than case-control studies. Second, in 1993, Sonnenberg and his colleagues performed a meta-analysis of fourteen case–control studies and reported that constipation increased the risk of colorectal cancer [31]. In 2013, Power also found that the prevalence of constipation in colorectal cancer patients was higher than that in the healthy controls (OR = 1.68; 95% CI: 1.29–2.18) [32]. In contrast, the included cohort studies in the current meta-analysis indicated that bowel movement frequency was associated with the risk of colorectal cancer. Additionally, the present study consisted of a large sample size of more than 1 million people. Third, previous studies treated constipation as an exposure factor. The definitions in different case-control studies were different, which may have affected the estimations. In the present study, we selected a specific index, namely, bowel movement frequency, as the exposure factor. We considered those with a frequency of once per day as the control group and those with a frequency of less than once per day and more than once per day as the exposure groups. Therefore, we ensured that all the exposure groups and non-exposure groups were consistent among the different studies. Finally, the previous studies did not perform subgroup analyses. In the present study, we extracted colon cancer and rectal cancer data and found that an increased frequency of bowel movements may be associated with elevated risk of rectal cancer but not colon cancer. Our meta-analysis has demonstrated new findings. Among all ten studies in our meta-analysis, the results were inconsistent. The EPIC-Norfolk study, which included 25,663 subjects aged 45–79 years, reported that loose stools could be an indicator of colorectal cancer risk, and the frequency of bowel movement was not associated with overall colorectal cancer risk [29]. In the Netherlands cohort study, Simons found that the frequency of bowel movements was not associated with the risk of colorectal cancer but increased the risk of rectal cancer (HR = 1.50, 95% CI: 1.15–1.95), and constipation was associated with decreased risks of colorectal cancer and rectal cancer [22]. This study’s findings were in accordance with our findings that the frequency of bowel movements increased the risk of rectal cancer. None of the other cohort studies, including three studies from the U.S.A. and one study considering a Japanese population, indicated that the frequency of bowel movements was related to the risk of colorectal cancer [23,27,28,30]. These studies consisted of large sample sizes, and the study populations were followed for more than 6 years. According to our results, the association between bowel movement and the colorectal cancer was affected by cancer type.

Previous studies suggested that constipation caused toxins to accumulate in the intestine over time, leading to the increased risk of colorectal cancer. The existing case–control studies also support this hypothesis. However, our results from the included cohort studies were in contrast with this hypothesis. We found a completely different result from those in previous research studies: the frequency of bowel movements may be associated with rectal cancer risk. It has also been reported that diarrhea and loose stools may stimulate the intestinal mucosa and induce the pathological proliferation of intestinal epithelial cells and abnormal intestinal epithelial structure and tumor formation. The production of irritants may be closely related to an imbalance in intestinal flora [33]. Experimental studies also reported that the level of prostaglandin E_2_ was significantly elevated in the gastrointestinal tract in people with diarrheal [34]. It was suggested that prostaglandin E_2_ can promote tumor growth by enhancing cell proliferation, prompting angiogenesis and inhibiting apoptosis [35,36]. This molecule was found to be involved in peristalsis *in vivo* [37]. Elevated prostaglandin E_2_ can inhibit the production and effects of some inflammatory factors, such as IL-2, thereby inhibiting T-cell-related tumor immunity mediated by IL-2, suppressing immunity to rectal cancer [38]. This may partly explain the molecular mechanisms.

The major strength of our study was the analysis of only cohort studies. A further strength was that the present studies consisted of more than 1 million study participants. Our study had several limitations. First, the bowel movement frequency was obtained from a self-reported questionnaire, and the reproducibility and validity of the responses were not confirmed. In addition, some recall bias may have existed. Second, the extracted data were from adjusted results, and the adjusted factors were different in some studies. Third, some studies also reported that some participants had taken laxatives before baseline data collection, potentially affecting the estimations.

Overall, our meta-analysis suggested that a bowel movement frequency of less than one time per day was not associated with the risk of colorectal cancer compared with a frequency of once per day. Compared with a bowel movement frequency of once per day, the frequency of bowel movements did not increase the risk of colon cancer but elevated the risk of rectal cancer. Due to the study number limitation in the subgroup analyses, further research is needed to confirm this result. However, as an easily recognized symptom, abnormal frequency of defecation should be emphasized in the practice of self-health management and early screening for rectal cancer.

## Supplementary Material

Supplementary Material S1-S6

## Abbreviations

CI: confidence internal
CRC: colorectal cancer
IL: interleukin
NOS: Newcastle–Ottawa Scale
RR: relative risk
