# Supplementary material for: Abnormal bowel movement frequency increases the risk of rectal cancer: evidence from cohort studies with one million people
Source: Biosci Rep. 2020 Apr 15;40(4):BSR20200355. doi: 10.1042/BSR20200355 (PMC7160378; doi:10.1042/BSR20200355)
Supplement: Supplementary Material S1-S6 [file BSR-2020-0355_supp.pdf]

Supplementary material 1 PRISMA Checklists

Section/topic	#	Checklist item	Reported on page #
**TITLE**			
Title	1	Identify the report as a systematic review, meta-analysis, or both.	Title
**ABSTRACT**			
Structured summary	2	Provide a structured summary including, as applicable: background; objectives; data sources; study eligibility criteria, participants, and interventions; study appraisal and synthesis methods; results; limitations; conclusions and implications of key findings; systematic review registration number.	Not applicable
**INTRODUCTION**			
Rationale	3	Describe the rationale for the review in the context of what is already known.	Introduction
Objectives	4	Provide an explicit statement of questions being addressed with reference to participants, interventions, comparisons, outcomes, and study design (PICOS).	Introduction
**METHODS**			
Protocol and registration	5	Indicate if a review protocol exists, if and where it can be accessed (e.g., Web address), and, if available, provide registration information including registration number.	Unpublished document circulated to collaborators
Eligibility criteria	6	Specify study characteristics (e.g., PICOS, length of follow-up) and report characteristics (e.g., years considered, language, publication status) used as criteria for eligibility, giving rationale.	Methods
Information sources	7	Describe all information sources (e.g., databases with dates of coverage, contact with study authors to identify additional studies) in the search and date last searched.	Methods
Search	8	Present full electronic search strategy for at least one database, including any limits used, such that it could be repeated.	Methods
Study selection	9	State the process for selecting studies (i.e., screening, eligibility, included in systematic review, and, if applicable, included in the meta-analysis).	Methods
Data collection process	10	Describe method of data extraction from reports (e.g., piloted forms, independently, in duplicate) and any processes for obtaining and confirming data from investigators.	Methods
Data items	11	List and define all variables for which data were sought (e.g., PICOS, funding sources) and any assumptions and simplifications made.	Methods
Risk of bias in individual studies	12	Describe methods used for assessing risk of bias of individual studies (including specification of whether this was done at the study or outcome level), and how this information is to be used in any data synthesis.	Methods
Summary measures	13	State the principal summary measures (e.g., risk ratio, difference in means).	Methods
Synthesis of results	14	Describe the methods of handling data and combining results of studies, if done, including measures of consistency (e.g., I^2) for each meta-analysis.	Methods

Section/topic	#	Checklist item	Reported on page #
Risk of bias across studies	15	Specify any assessment of risk of bias that may affect the cumulative evidence (e.g., publication bias, selective reporting within studies).	Results
Additional analyses	16	Describe methods of additional analyses (e.g., sensitivity or subgroup analyses, meta-regression), if done, indicating which were pre-specified.	Methods<br>Results
**RESULTS**			
Study selection	17	Give numbers of studies screened, assessed for eligibility, and included in the review, with reasons for exclusions at each stage, ideally with a flow diagram.	Methods<br>Figure 1
Study characteristics	18	For each study, present characteristics for which data were extracted (e.g., study size, PICOS, follow-up period) and provide the citations.	Table 1
Risk of bias within studies	19	Present data on risk of bias of each study and, if available, any outcome level assessment (see item 12).	Figure 2-6
Results of individual studies	20	For all outcomes considered (benefits or harms), present, for each study: (a) simple summary data for each intervention group (b) effect estimates and confidence intervals, ideally with a forest plot.	Figure2-6
Synthesis of results	21	Present results of each meta-analysis done, including confidence intervals and measures of consistency.	Table 2
Risk of bias across studies	22	Present results of any assessment of risk of bias across studies (see Item 15).	Figure 7
Additional analysis	23	Give results of additional analyses, if done (e.g., sensitivity or subgroup analyses, meta-regression [see Item 16]).	Table 2
**DISCUSSION**			
Summary of evidence	24	Summarize the main findings including the strength of evidence for each main outcome; consider their relevance to key groups (e.g., healthcare providers, users, and policy makers).	Discussion
Limitations	25	Discuss limitations at study and outcome level (e.g., risk of bias), and at review-level (e.g., incomplete retrieval of identified research, reporting bias).	Discussion
Conclusions	26	Provide a general interpretation of the results in the context of other evidence, and implications for future research.	Discussion
**FUNDING**			
Funding	27	Describe sources of funding for the systematic review and other support (e.g., supply of data); role of funders for the systematic review.	Declared on online submission system

Supplementary material 2 Search strategy and Search terms

1. PubMed (n=965):

((((((((((bowel movement[MeSH Terms])) OR (bowel movement frequency[MeSH Terms])) OR (bowel habit[MeSH Terms])) OR (constipation[MeSH Terms])) OR (functional constipation[MeSH Terms])) OR (idiopathic constipation[MeSH Terms])) OR (chronic constipation[MeSH Terms])) OR (slow transit[MeSH Terms])) OR (state of stool[MeSH Terms]))) AND ((((((((((((((((colon cancer[MeSH Terms])) OR (rectal cancer[MeSH Terms])) AND (colorectal cancer[MeSH Terms])) OR (bowel cancer[MeSH Terms])) OR (colon carcinoma[MeSH Terms])) OR (rectal carcinoma[MeSH Terms])) OR (colorectal carcinoma[MeSH Terms])) OR (bowel carcinoma[MeSH Terms])) OR (colon adenocarcinoma[MeSH Terms])) OR (rectal adenocarcinoma[MeSH Terms])) OR (colorectal adenocarcinoma[MeSH Terms])) OR (bowel adenocarcinoma[MeSH Terms])) OR (colonic neoplasms[MeSH Terms])) OR (rectal neoplasms[MeSH Terms])) OR (colorectal neoplasms[MeSH Terms])) OR (CRC[MeSH Terms]))

2. Web of Science (n=1415):

Step 1: TOPIC: (colon cancer) OR TOPIC: (rectal cancer) OR TOPIC: (colorectal cancer) OR TOPIC: (bowel cancer) OR TOPIC: (colon carcinoma) OR TOPIC: (rectal carcinoma) OR TOPIC: (colorectal carcinoma) OR TOPIC: (bowel carcinoma) OR TOPIC: (colon adenocarcinoma) OR TOPIC: (rectal adenocarcinoma) OR TOPIC: (colorectal adenocarcinoma) OR TOPIC: (bowel adenocarcinoma) OR TOPIC: (colonic neoplasms) OR TOPIC: (rectal neoplasms) OR TOPIC: (colorectal neoplasms) OR TITLE: (CRC)

Databases= WOS, BCI, KJD, MEDLINE, RSCI, SCIELO Timespan=All years

Search language=Auto

Step 2: TOPIC: (bowel movement) OR TOPIC: (bowel movement frequency) OR TOPIC: (constipation) OR TOPIC: (functional constipation) OR TOPIC: (idiopathic constipation) OR TOPIC: (chronic constipation) OR TOPIC: (slow transit) OR TOPIC: (state of stool) OR TOPIC: (stool state)

Databases= WOS, BCI, KJD, MEDLINE, RSCI, SCIELO Timespan=All years

Search language=Auto

Step 3: TOPIC: (cohort study) OR TOPIC: (prospective cohort study) OR TOPIC: (retrospective cohort study) OR TOPIC: (prospective) OR TOPIC: (retrospective) OR TOPIC: (follow-up)

Databases= WOS, BCI, KJD, MEDLINE, RSCI, SCIELO Timespan=All years

Search language=Auto

Step 4: #4 AND #2 AND #1

Databases= WOS, BCI, KJD, MEDLINE, RSCI, SCIELO Timespan=All years

Search language=Auto

3. Embase(n=3231):

Step 1: 'colon cancer':ti OR 'rectal cancer':ti OR 'colorectal cancer':ti OR 'bowel cancer'/exp OR 'bowel cancer' OR (('bowel'/exp OR bowel) AND ('cancer'/exp OR cancer)) OR 'colon carcinoma'/exp OR 'colon carcinoma' OR (('colon'/exp OR colon) AND ('carcinoma'/exp OR carcinoma)) OR 'rectal carcinoma'/exp OR 'rectal carcinoma' OR (rectal AND ('carcinoma'/exp OR carcinoma)) OR 'colorectal carcinoma'/exp OR 'colorectal carcinoma' OR (colorectal AND ('carcinoma'/exp OR carcinoma)) OR 'bowel carcinoma'/exp OR 'bowel carcinoma' OR (('bowel'/exp OR bowel) AND ('carcinoma'/exp OR carcinoma)) OR 'colon adenocarcinoma'/exp OR 'colon adenocarcinoma' OR (('colon'/exp OR colon) AND ('adenocarcinoma'/exp OR adenocarcinoma)) OR 'rectal adenocarcinoma'/exp OR 'rectal adenocarcinoma' OR (rectal AND ('adenocarcinoma'/exp OR adenocarcinoma)) OR 'colorectal adenocarcinoma'/exp OR 'colorectal adenocarcinoma' OR (colorectal AND ('adenocarcinoma'/exp OR adenocarcinoma)) OR 'bowel adenocarcinoma' OR (('bowel'/exp OR bowel) AND ('adenocarcinoma'/exp OR adenocarcinoma)) OR 'colonic neoplasms'/exp OR 'colonic neoplasms' OR (colonic AND ('neoplasms'/exp OR neoplasms)) OR 'rectal neoplasms'/exp OR 'rectal neoplasms' OR (rectal AND ('neoplasms'/exp OR neoplasms)) OR 'colorectal neoplasms'/exp OR 'colorectal neoplasms' OR (colorectal AND ('neoplasms'/exp OR neoplasms))

Step 2: 'bowel movement'/exp OR 'bowel movement' OR (('bowel'/exp OR bowel) AND ('movement'/exp OR movement)) OR 'bowel movement frequency' OR (('bowel'/exp OR bowel) AND ('movement'/exp OR movement) AND ('frequency'/exp OR frequency)) OR 'bowel habit'/exp OR 'bowel habit' OR (('bowel'/exp OR bowel) AND ('habit'/exp OR habit)) OR 'constipation'/exp OR constipation OR 'functional constipation'/exp OR 'functional

constipation' OR (functional AND ('constipation'/exp OR constipation)) OR 'idiopathic constipation' OR (idiopathic AND ('constipation'/exp OR constipation)) OR 'chronic constipation'/exp OR 'chronic constipation' OR (chronic AND ('constipation'/exp OR constipation)) OR 'slow transit' OR (slow AND transit) OR 'stool state' OR (('stool'/exp OR stool) AND ('state'/exp OR state))

Step 3: 'cohort study'/exp OR 'cohort study' OR (cohort AND ('study'/exp OR study)) OR 'prospective cohort study' OR (prospective AND cohort AND ('study'/exp OR study)) OR 'retrospective cohort study' OR (retrospective AND cohort AND ('study'/exp OR study)) OR prospective OR retrospective OR 'follow up'/exp OR 'follow up'

Step 4: #1 AND #2 AND #4

4.CNKI and Wanfang (n=134):

（结直肠癌 OR 结肠癌 OR 直肠癌 OR 结直肠肉瘤 OR 结肠肉瘤 OR 直肠肉瘤）

AND （便秘 OR 肠道运动 OR 肠道运动频率 OR 肠运动 OR 肠运动频率）AND

（队列研究 OR 回顾性研究 OR 流行病学 OR 随访）

Supplemental material 3 List of excluded references and reasons for exclusion

NO.	References	Reason
1	Tashiro N, Budhathoki S, Ohnaka K, Toyomura K, Kono S, Ueki T, Tanaka M, Kakeji Y, Maehara Y, Okamura T, Ikejiri K, Futami K, Maekawa T, Yasunami Y, Takenaka K, Ichimiya H, Terasaka R. Constipation and colorectal cancer risk: the Fukuoka Colorectal Cancer Study. Asian Pac J Cancer Prev.2011;12(8):2025-2030.	Case-control
2	Kobayashi T, Masaki T, Kogawa K, Matsuoka H, Sugiyama M. Efficacy of Gum Chewing on Bowel Movement After Open Colectomy for Left-Sided Colorectal Cancer: A Randomized Clinical Trial. Dis Colon Rectum.2015;58(11):1058-1063.	Unrelated topic
3	Ohara T, Yoshino K, Kitajima M. [Pre- and probiotics increase host-cell immunological competence, improve bowel movement, and prevent the onset of colon cancer-an analysis based on movements of intestinal microbiota]. Rinsho Byori.2009;57(6):533-541.	Unrelated topic
4	Talley NJ. Editorial: adequate management may reduce the colorectal cancer risk associated with constipation? Author's reply. Aliment Pharmacol Ther.2014;40(5):564-565.	Editorial
5	Scarpignato C, Blandizzi C. Editorial: adequate management may reduce the colorectal cancer risk associated with constipation. Aliment Pharmacol Ther.2014;40(5):562-564.	Editorial
6	Power AM, Talley NJ, Ford AC. Association between constipation and colorectal cancer: systematic review and meta-analysis of observational studies. Am J Gastroenterol.2013;108(6):894-903, 904.	Systematic review
7	Tashiro N, Budhathoki S, Ohnaka K, Toyomura K, Kono S, Ueki T, Tanaka M, Kakeji Y, Maehara Y, Okamura T, Ikejiri K, Futami K, Maekawa T, Yasunami Y, Takenaka K, Ichimiya H, Terasaka R. Constipation and colorectal cancer risk: the Fukuoka Colorectal Cancer Study. Asian Pac J Cancer Prev.2011;12(8):2025-2030.	Duplicates
8	Tayyem RF, Shehadeh IN, Abumweis SS, Bawadi HA, Hammad SS, Bani-Hani KE, Al-Jaberi TM, Alnusair MM. Physical inactivity, water intake and constipation as risk factors for colorectal cancer among adults in Jordan. Asian Pac J Cancer Prev.2013;14(9):5207-5212.	Case-control
9	Chan AO, Hui WM, Leung G, Tong T, Hung IF, Chan P, Hsu A, But D, Wong BC, Lam SK, Lam KF. Patients with functional constipation do not have increased prevalence of colorectal cancer precursors. Gut.2007;56(3):451-452.	Case-Control
10	Guerin A, Mody R, Fok B, Lasch KL, Zhou Z, Wu EQ, Zhou W, Talley NJ. Risk of developing colorectal cancer and benign colorectal neoplasm in patients with chronic constipation. Aliment Pharmacol Ther.2014;40(1):83-92.	Duplicates
11	Shemerovskii KA. [Constipation--a risk factor for colorectal cancer]. Klin Med (Mosk).2005;83(12):60-64.	No data extraction

12	Kune GA, Kune S, Field B, Watson LF. The role of chronic constipation, diarrhea, and laxative use in the etiology of large-bowel cancer. Data from the Melbourne Colorectal Cancer Study. Dis Colon Rectum.1988;31(7):507-512.	Case-Control
13	Sonnenberg A, Muller AD. Constipation and cathartics as risk factors of colorectal cancer: a meta-analysis. Pharmacology.1993;47 Suppl 1:224-233.	Meta-analysis
14	Tibana TK, Santos R, Marques P, Marchiori E, Nunes TF. Obstructive colorectal cancer presenting as constipation during pregnancy. Radiol Bras.2019;52(3):207-208	Special population
15	Abraham JM, Taylor CJ. Cystic Fibrosis & disorders of the large intestine: DIOS, constipation, and colorectal cancer. J Cyst Fibros.2017;16 Suppl 2:S40-S49.	Systematical review
16	Furst A. [Differential indications for ileoanal pouch anastomosis : Ulcerative colitis, familial adenomatous polyposis, synchronous colorectal cancer - Crohn's disease, constipation]. Chirurg.2017;88(7):555-558.	Case-control
17	Keely S, Veysey M, Walker MM, Talley NJ. Letter: oxidative stress, cause or consequence of constipation-associated colorectal cancer? Aliment Pharmacol Ther.2015;42(7):941-942.	Letter
18	Jones A, Povlow MR. Colorectal Cancer Presenting with Constipation During Pregnancy. Cureus.2017;9(4):e1190.	Case report
19	Anderson JC, Lacy BE. Editorial: Constipation and colorectal cancer risk: a continuing conundrum. Am J Gastroenterol.2014;109(10):1650-1652.	Editorial
20	Lee GY, Lee SM, Jang JH, Oh HK, Kim DW, Ahn S, Kang SB. Preoperative constipation is associated with poor prognosis of rectal cancer: a prospective cohort study. J Korean Surg Soc.2013;85(1):35-42.	Prognosis analysis
21	Le Marchand L. Constipation and colon cancer. Epidemiology.1998;9(4):371-372.	Comment
22	Jacobs EJ, White E. Constipation, laxative use, and colon cancer among middle-aged adults. Epidemiology.1998;9(4):385-391.	Case-control
23	Nascimbeni R, Donato F, Ghirardi M, Mariani P, Villanacci V, Salerni B. Constipation, anthranoid laxatives, melanosis coli, and colon cancer: a risk assessment using aberrant crypt foci. Cancer Epidemiol Biomarkers Prev.2002;11(8):753-757.	Case-control
24	Roberts MC, Millikan RC, Galanko JA, Martin C, Sandler RS. Constipation, laxative use, and colon cancer in a North Carolina population. Am J Gastroenterol.2003;98(4):857-864.	Case-control

Supplementary material 4 Methodological quality assessment (risk of bias) of included studies by Newcastle-Ottawa Scales

**Study**	**Selection**				**Comparability**	**Outcomes**			**Total score**
	Exposed cohort	Nonexposed cohort	Ascertainment of exposure	Outcomes of interest		Assessment of outcome	Length of follow-up	Adequate of follow-up	
Nakaya	*	*	*	*	**	*	*	*	9
Park	*	-	*	*	*	*	*	*	7
Otani	*	*	*	*	**	*	*	*	9
Kojima	*	*	*	*	*	*	*	*	8
Citronberg	*	*	*	*	**	*	*	*	9
Simons	*	*	*	*	*	*	*	*	8
Yang	*	*	*	*	**	*	*	*	9
Zhang	*	*	*	*	*	*	*	*	8
Dukas	*	*	*	*	*	*	*	*	8
Guerin	*	*	*	*	*	*	*	*	8

Supplementary material 5 Pooling results about the relationship between bowel movement and colorectal cancer risk basing on fixed-effect and random-effects model

Category	Group	Fixed-effect model			Random-effects model		
		RR	**95%CI**	P	RR	**95%CI**	P
**Unadjusted**							
<1 time per day	Colorectal cancer	0.98	0.93-1.03	0.410	0.87	0.69-1.09	0.220
	Colon cancer	0.82	0.76-0.89	0.000	0.80	0.72-0.89	0.000
	Rectal cancer	0.73	0.64-0.82	0.000	0.74	0.66-0.83	0.000
>1 time per day	Colorectal cancer	1.12	0.05-1.19	0.000	1.03	0.88-1.20	0.740
	Colon cancer	1.11	1.01-1.22	0.030	1.12	1.01-1.23	0.020
	Rectal cancer	1.45	1.29-1.62	0.000	1.39	1.16-1.67	0.000
**Adjusted**							
<1 time per day	Colorectal cancer				1.00	0.87-1.16	0.950
	Colon cancer	0.91	0.80-1.03	0.130	0.93	0.79-1.11	0.720
	Rectal cancer	0.93	0.78-1.11	0.420	0.93	0.78-1.11	0.420
>1 time per day	Colorectal cancer	1.09	1.02-1.17	0.010	1.04	0.91-1.19	0.570
	Colon cancer	1.04	0.92-1.18	0.054	1.04	0.92-1.18	0.540
	Rectal cancer	1.34	1.19-1.52	0.000	1.31	1.11-1.56	0.002

Supplementary material 6 Sensitivity analysis of pooling results about the relationship between bowel movement and colorectal cancer risk

**Category**	**Group**	**Study omitted**	**RR**	**LCI**	**UCI**	**P**
**Unadjusted**						
<1 time per day	Colorectal cancer	**Overall**	0.87	0.69	1.09	0.220
		Citrongber, 2014	0.85	0.66	1.10	0.220
		Dukas, 2000	0.87	0.67	1.11	0.260
		Guerin, 2014	0.70	0.74	0.88	0.000
		Nakaya, 2004	0.86	0.67	1.09	0.210
		Otani1, 2006	0.89	0.70	1.13	0.330
		Park, 2009	0.87	0.69	1.10	0.250
		Simons1, 2010	0.88	0.69	1.12	0.310
		Wakai1,2004	0.90	0.71	1.14	0.370
		Yang, 2019	0.87	0.67	1.13	0.300
		Zhang1,2013	0.87	0.66	1.13	0.290
		Zhang1, 2013	0.87	0.69	1.11	0.270
	Colon cancer	**Overall**	0.80	0.72	0.89	0.000
		Dukas, 2000	0.78	0.68	0.89	0.000
		Nakaya, 2004	0.82	0.73	0.91	0.000
		Otani1, 2006	0.81	0.72	0.91	0.000
		Simons1, 2010	0.79	0.69	0.89	0.000
		Wakai1,2004	0.85	0.79	0.92	0.000
		Yang, 2019	0.78	0.68	0.90	0.001
		Zhang1,2013	0.77	0.68	0.87	0.000
		Zhang1, 2013	0.80	0.71	0.90	0.000
	Rectal cancer	**Overall**	0.74	0.66	0.83	0.000
		Dukas, 2000	0.73	0.64	0.74	0.000
		Nakaya, 2004	0.73	0.65	0.83	0.000
		Otani1, 2006	0.73	0.64	0.84	0.000
		Simons1, 2010	0.75	0.66	0.84	0.000
		Wakai1,2004	0.75	0.66	0.84	0.000
		Yang, 2019	0.67	0.56	0.79	0.000
		Zhang1,2013	0.77	0.67	0.88	0.000
		Zhang1, 2013	0.73	0.64	0.84	0.000
>1 time per day	Colorectal cancer	**Overall**	1.03	0.88	1.20	0.740
		Citrongber, 2014	1.04	0.87	1.24	0.650
		Dukas, 2000	1.05	0.89	1.24	0.590
		Otain1, 2006	1.04	0.87	1.23	0.700
		Simons1. 2010	0.99	0.81	1.21	0.910
		Yang, 2019	0.97	0.85	1.12	0.700
		Zhang1, 2013	1.06	0.90	1.24	0.500
		Zhang2. 2013	1.05	0.89	1.24	0.560
	Colon cancer	Overall	1.12	1.01	1.23	0.020
		Dukas, 2000	1.14	1.03	1.25	0.010
		Otain1, 2006	1.11	1.00	1.23	0.050
		Simons1, 2010	1.15	1.04	1.28	0.008
		Simons2, 2010	1.11	1.01	1.23	0.040

		Simons3, 2010	1.09	0.98	1.21	0.120
		Simons4, 2010	1.12	1.02	1.24	0.020
		Yang, 2019	1.06	0.94	1.20	0.300
	Rectal cancer	**Overall**	1.39	1.16	1.67	0.000
		Dukas, 2000	1.50	1.33	1.68	0.000
		Otain1,2006	1.36	1.08	1.71	0.009
		Simons1, 2010	1.30	0.97	1.74	0.080
		Simons2, 2010	1.38	1.12	1.71	0.003
		Yang, 2019	1.27	0.97	1.66	0.090
**Adjusted**						
<1 time per day	Colorectal cancer	**Overall**	1.00	0.87	1.16	0.950
		Citrongber,2014	1.00	0.85	1.17	0.990
		Dukas, 2000	1.01	0.86	1.19	0.890
		Guerin, 2014	0.95	0.84	1.08	0.460
		Nakaya, 2004	0.98	0.84	1.15	0.840
		Otain1, 2006	1.01	0.87	1.18	0.860
		Otain2, 2006	1.02	0.88	1.19	0.760
		Parker, 2009	0.98	0.84	1.13	0.740
		Simons1, 2004	1.03	0.88	1.20	0.710
		Wakai1, 2004	1.00	0.86	1.17	0.960
		Wakai2, 2004	0.98	0.85	1.14	0.830
		Wakai3, 2004	1.01	0.87	1.17	0.880
		Wakai4, 2004	1.00	0.86	1.17	0.990
		Yang, 2019	1.03	0.88	1.20	0.730
		Zhang1, 2013	1.03	0.88	1.20	0.760
		Zhang2, 2013	1.02	0.87	1.18	0.830
	Colon cancer	**Overall**	0.91	0.80	1.03	0.130
		Dukas, 2000	0.91	0.80	1.03	0.150
		Guerin, 2014	0.91	0.80	1.04	0.170
		Nakaya, 2004	0.86	0.75	0.98	0.020
		Otain1,2006	0.90	0.79	1.02	0.090
		Otain2, 2004	0.93	0.82	1.05	0.240
		Wakai1.2004	0.90	0.80	1.03	0.120
		Wakai2,2004	0.89	0.79	1.01	0.080
		Wakai3, 2004	0.90	0.80	1.03	0.120
		Yang, 2019	0.91	0.80	1.04	0.160
		Zhang1, 2013	0.95	0.82	1.09	0.440
		Zhang2, 2013	0.93	0.80	1.08	0.350
	Rectal cancer	**Overall**	0.93	0.78	1.11	0.420
		Dukas, 2000	0.91	0.76	1.10	0.330
		Nakaya, 2004	0.91	0.75	1.09	0.290
		Otain1, 2006	0.94	0.79	1.13	0.051
		Otain2, 2006	0.93	0.77	1.11	0.410
		Simons1, 2010	0.95	0.79	1.14	0.59
		Yang, 2019	0.93	0.74	1.16	0.500
		Zhang1, 2013	0.95	0.78	1.16	0.610
		Zhang2, 2013	0.94	0.78	1.12	0.470

>1 time per day	Colorectal cancer	**Overall**	1.04	0.91	1.19	0.054
		Citrongber,2014	1.06	0.92	1.22	0.450
		Dukas, 2000	1.07	0.93	1.22	0.340
		Otain1,2006	1.04	0.90	1.20	0.610
		Otain2, 2006	1.03	0.90	1.19	0.660
		Simons1, 2010	1.03	0.89	1.18	0.720
		Simons2, 2010	1.00	0.87	1.16	0.970
		Yang, 2019	1.00	0.87	1.15	0.980
		Zhang1, 2013	1.08	0.95	1.23	0.220
		Zhang2, 2013	1.05	0.90	1.21	0.540
	Colon cancer	**Overall**	1.04	0.92	1.18	0.540
		Dukas, 2000	1.07	0.93	1.24	0.310
		Otain1,2006	1.05	0.92	1.20	0.470
		Otain2, 2006	1.04	0.92	1.19	0.520
		Simons1, 2010	1.04	0.91	1.18	0.570
		Simons2, 2010	1.05	0.92	1.19	0.470
		Yang, 2019	0.92	0.75	1.13	0.430
	Rectal cancer	**Overall**	1.31	1.11	1.56	0.002
		Dukas, 2000	1.38	1.22	1.56	0.000
		Otain1,2006	1.31	1.06	1.61	0.010
		Otain2, 2006	1.28	1.05	1.56	0.010
		Simons1, 2010	1.30	1.06	1.59	0.010
		Simons2, 2010	1.22	0.94	1.57	0.130
		Yang, 2019	1.22	0.91	1.64	0.190
